# Laparoscopic Management of Boerhaave Syndrome: Case Series and Narrative Review

**DOI:** 10.3390/life15121865

**Published:** 2025-12-05

**Authors:** Floris Cristian Stănculea, Andrei Razvan Stoica, Claudiu Octavian Ungureanu, Raul Mihailov, Octav Ginghina, Mircea Litescu, Niculae Iordache, Nicoleta Alina Mares, Alexandru Iordache

**Affiliations:** 1 Carol Davila, General Medicine Faculty, University of Medicine and Pharmacy, 37 Dionisie Lupu Street, 020021 Bucharest, Romania; floris-cristian.stanculea@drd.umfcd.ro (F.C.S.); andrei.stoica@umfcd.ro (A.R.S.); octav.ginghina@umfcd.ro (O.G.); mircea.litescu@umfcd.ro (M.L.); alinamaresh@yahoo.com (N.A.M.); 2 General Surgery Department, “Sfantul Ioan” Clinical Emergency Hospital, 13 Vitan-Bârzeşti Road, 042122 Bucharest, Romania; 3 General Surgery Department, “Sfantul Andrei” Clinical Emergency Hospital, 177 Braila Street, 800578 Galati, Romania; raulmihailov@yahoo.com; 4 Department of General Surgery, Prof. Dr. Al. Trestioreanu Oncological Institute, 252 Fundeni Street, 022328 Bucharest, Romania; 5 The Academy of Romanian Scientists, Ilfov 3 Street, 030167 Bucharest, Romania; 6 “Professor Doctor Theodor Burghele” Clinical Hospital, 20 Panduri Road, 050659 Bucharest, Romania; alexandru.ioradche@drd.umfcd.ro

**Keywords:** esophagus, Boerhaave syndrome, rupture, spontaneous, laparoscopic, emergency

## Abstract

Boerhaave syndrome, or spontaneous rupture of the esophageal wall, is a rare condition that occurs after a violent increase in intraesophageal pressure associated with negative intrathoracic pressure after forceful vomiting or retching. Rupture, usually transmural, often occurs in the distal esophagus and can be complicated by mediastinitis, sepsis, and multiple organ dysfunction. Despite the aid of imaging in diagnosis and rapid management, it is still characterized by a high mortality rate. Surgical and endoscopic approaches are the mainstays of treatment. Case presentation: Here, we describe three cases of Boerhaave’s syndrome managed with minimally invasive surgical treatment and provide a brief literature review of the treatment methods for this rare condition. Conclusions: Early diagnosis and prompt management by a multidisciplinary team increase the chances of survival. The laparoscopic approach is beneficial and can successfully treat esophageal tears associated with this syndrome in early or delayed timing.

## 1. Introduction

Since its initial identification by Boerhaave in 1724, spontaneous esophageal perforation has consistently been characterized in the medical literature as a critical and life-threatening condition requiring urgent attention [1]. It has been reported that only 15% of esophageal perforations are spontaneous [2]. The most identified risk factors are excessive alcohol consumption and episodes of overeating, with a predominant male distribution and incidence in the population aged 50–70 years [3,4]. Furthermore, a variety of other precipitating causes have been recognized for their potential to initiate the pathophysiological cascade leading to Boerhaave syndrome (BS), including abdominal trauma, seizure activity, heavy lifting, straining during defecation, and, notably, childbirth in women [3,5]. In approximately 80% of cases, elevated intraesophageal pressure leads to full-thickness tears of the distal esophageal wall. This rupture most commonly occurs along the left posterolateral aspect, approximately 3–6 cm proximal to the diaphragmatic hiatus. This predilection is thought to result from a combination of anatomical and structural factors [5,6,7,8,9,10]. Successful management of this condition largely depends on prompt recognition. Outcomes are most favorable when perforation is identified within the first 12–24 h. The initial therapeutic strategy encompasses strict cessation of oral intake, institution of parenteral nutritional support, administration of broad-spectrum antimicrobial therapy, suppression of gastric acid secretion with proton pump inhibitors, and adequate drainage of fluid collections or abscesses [11]. Definitive management of esophageal perforations may be undertaken through surgical or endoscopic repair, with the selected modality determined by the anatomical site and extent of the defect, the patient’s overall physiological status and comorbidities, and, critically, the interval between diagnosis and initiation of intervention [12]. The mortality rate of BS ranges from 20 to 30%, as some authors have reported [13], to 40–50% as others have claimed [14].

## 2. Materials and Methods

We present three cases of early presentation of BS that were managed using a surgical laparoscopic approach. Table 1 summarizes the main features of these cases. Laboratory tests are often nonspecific in the diagnosis of Boerhaave syndrome, and our data are no exception.

### Surgical Technique

In all cases, a laparoscopic transhiatal technique was used. The patient was placed in the supine position with the legs in the modified lithotomy position in the reverse Trendelenburg position. We used a setup based on five trocars, as follows: an optical trocar (10 mm) to gain access to the abdomen, usually left to the midline, situated 10–15 cm from the xiphisternum; an upper left quadrant (5 mm) and an upper right quadrant trocar (10 mm), close to the costal margin, for the working ports; a left flank (5 mm) trocar for the retraction by the assistant; and a liver retractor port (5 mm) placed at the xiphisternum. Definitive repair was performed through a single-layer closure of both the mucosa and muscularis using continuous absorbable 3-0 sutures; we routinely reinforced with an omental patch.

## 3. Cases Presentations

### 3.1. Case 1

#### 3.1.1. Presentation

We present the case of a 54-year-old male patient, residing in an urban environment, with a history of hypertension and habitual drinking, who presented in our emergency room with acute onset from 6 h till presentation of unbearable epigastric and retrosternal pain after severe emesis, followed by hematemesis and forceful retching. At presentation, we found 125/87 a blood pressure, a regular heart rate of 87 beats per minute, a body temperature of 36.7 °C, a respiratory rate of 23 breaths per minute, and an oxygen saturation level of 92%. His height and weight were 173 cm and 73.1 kg, respectively. Physical examination at presentation revealed a conscious, anxious patient with altered general status, pale skin, sweating, retrosternal pain associated with unbearable epigastric pain, and cold extremities.

#### 3.1.2. Diagnosis

**Figure 1:**
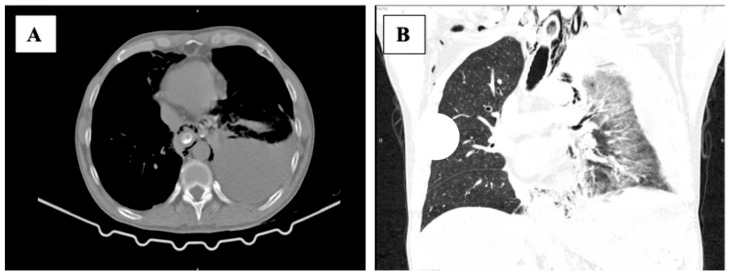
(**A**) Transverse CT scan of the chest, revealing moderate pneumomediastinum, especially in the posterior mediastinum. (**B**) Longitudinal CT section indicates left pleural effusion.

A series of investigations was performed to establish a definitive diagnosis. CT (computed tomography) revealed several pathological features, including the presence of moderate pneumomediastinum, predominantly localized within the posterior mediastinum (Figure 1A), along with a discernible fistulous tract arising from the lateral wall of the distal esophagus, which also demonstrated mural thickening and edema associated with pleural effusion. Following oral contrast administration, extraluminal leakage of the contrast medium into the mediastinum at the level of the distal esophagus was detected (Figure 1B), which was highly suggestive of BS.

**Figure 2:**
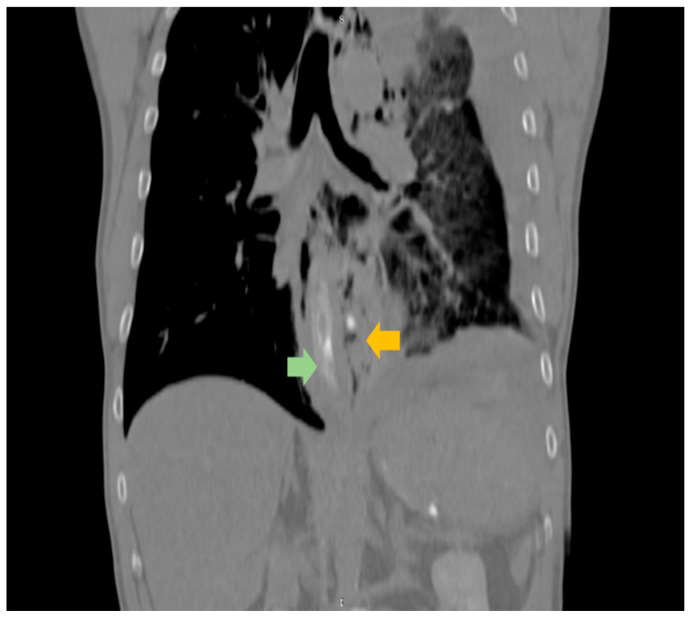
Longitudinal CT sections obtained after oral contrast administration demonstrated extraluminal leakage of the contrast material into the mediastinum at the distal esophagus level. The distal esophageal segment appeared thickened and edematous (green arrow), and the site of perforation was visualized as the point of contrast escape from the esophageal lumen (orange arrow).

The patient was admitted to the general surgery department, associated with the thoracic surgery section, with a working diagnosis of BS complicated by acute mediastinitis. Given that the esophageal rupture was situated in the distal third of the esophagus (Figure 2) and that the patient presented within only a few hours of symptom onset, prompt operative management was deemed the appropriate course of action.

#### 3.1.3. Operative Details

**Figure 3:**
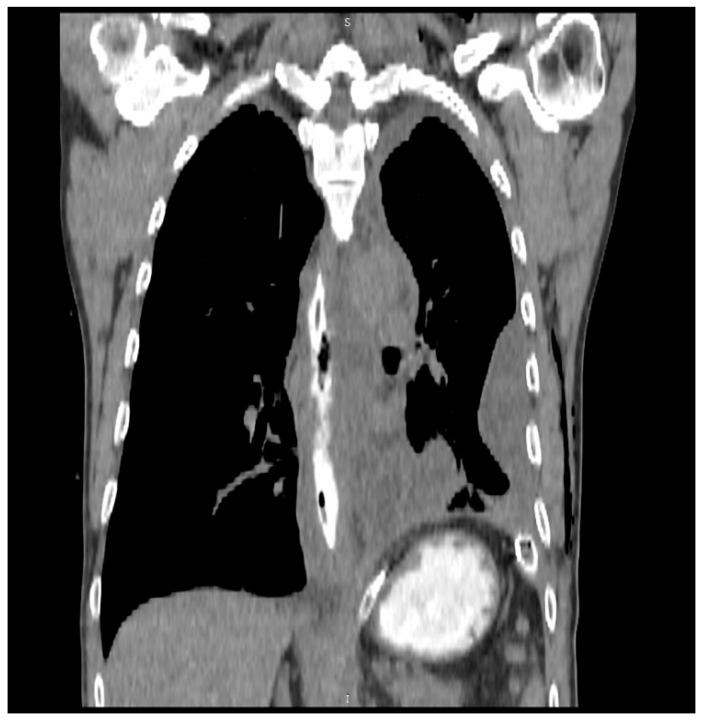
Follow-up longitudinal CT performed 10 days after surgery revealed favorable postoperative evolution. The esophagus appeared intact, without evidence of fistulous tracts, and there were no signs of gastroesophageal reflux or hiatal hernia. The cardia was patent, and the stomach maintained a normal tone and active peristalsis, with supple, well-preserved mucosal folds.

Therefore, emergency surgery was performed, consisting of primary laparoscopic transhiatal mobilization and suture repair of the esophageal perforation, thorough mediastinal drainage to control contamination, and placement of a laparoscopic feeding jejunostomy to ensure adequate nutritional support during the postoperative period. Following thorough debridement of the contaminated fluid and residual food material in the vicinity of the esophageal rupture, the exact site of mucosal disruption was identified and marked. To achieve optimal exposure, longitudinal myotomy was performed to visualize the esophageal defect. The lesion was located on the left lateral wall of the distal thoracic esophagus, immediately above the gastroesophageal junction, and measured approximately 30 mm in length. Definitive repair was performed through a single-layer closure of both the mucosa and muscularis using continuous absorbable 3-0 sutures. The repair site was reinforced with an omental patch to provide additional support. Finally, a drain was placed in the lower mediastinum via the diaphragmatic hiatus to ensure adequate postoperative drainage of the abdominal cavity. Immediately after the intervention, the patient was transferred to the intensive care unit (ICU) for hemodynamic monitoring, respiratory support, and comprehensive postoperative care. Follow-up longitudinal CT performed 10 days after surgery revealed an intact esophagus without evidence of a fistula (Figure 3).

In the postoperative course, the patient showed steady improvement, which allowed for discharge on the 19th postoperative day with a functional jejunostomy for nutritional support.

#### 3.1.4. Follow-Up

Follow-up evaluations indicated continued favorable recovery, and the jejunostomy was successfully removed 40 days postoperatively. At 60 days after the surgical intervention, a barium swallow study and upper digestive endoscopy were performed, which confirmed favorable postoperative evolution. The esophagus appeared radiologically normal, with no evidence of gastroesophageal reflux or hiatal hernia. The cardia was patent, and the stomach maintained a normal tone and contractility, displaying supple and regular mucosal folds. The pyloric orifice was unobstructed, and the duodenal bulb and frame exhibited a normal radiological configuration.

### 3.2. Case 2

#### 3.2.1. Presentation

A 38-year-old man, previously in good health, presented to our emergency department with complaints of vomiting, hematemesis, heartburn, intense chest pain, and epigastric discomfort. He had consumed large amounts of alcohol for approximately eight hours before admission. About four hours prior to arrival, he began experiencing epigastric pain and nausea, which were soon followed by repeated retching. Roughly two and a half hours before presentation, heartburn and intermittent retrosternal pain developed, culminating in an episode of hematemesis. On admission, the patient was alert and hemodynamically stable, with a blood pressure of 122/64 mmHg, pulse of 96 bpm, temperature of 37.9 °C, respiratory rate of 22/min, and oxygen saturation of 96%. His height and weight were 184 cm and 75 kg, respectively. Physical examination revealed epigastric tenderness and retrosternal pain exacerbated by deep inspiration.

#### 3.2.2. Diagnosis

Although the chest radiograph appeared normal, contrast-enhanced CT revealed small collections of mediastinal free air. Initially, Mallory–Weiss syndrome was considered due to the episode of hematemesis; however, the CT findings raised suspicion for BS. The patient was initially managed conservatively with nil per os, intravenous broad-spectrum antibiotics, and mediastinal drainage. However, owing to persistent signs of mediastinal contamination and a lack of clinical improvement, surgical intervention was deemed necessary.

#### 3.2.3. Operative Details

**Figure 4:**
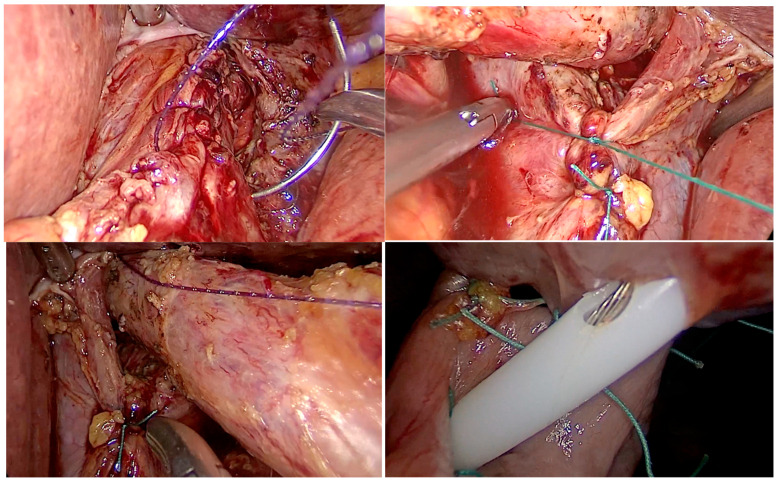
Intraoperative aspects of laparoscopic management of Boerhaave’s syndrome. **Top left** image: suture of the esophageal tear; **top right** image: suture of crura; **bottom left** image: aspect of the hiatus after crura calibration; **bottom right** image: jejunostomy technique details.

On the fifth day of hospitalization, a laparoscopic transhiatal approach was used to explore and repair the esophageal perforation. Intraoperative findings confirmed a linear perforation in the left wall of the distal esophagus, near the gastroesophageal junction (Figure 4). The defect was primarily closed with absorbable sutures in a single layer, and the repair site was reinforced with an omental patch. A mediastinal drain was placed through the hiatus to ensure adequate postoperative mediastinal drainage. Following surgery, the patient’s condition gradually improved, allowing for a progressive return to oral intake, and he was discharged on the 14th postoperative day in good general condition.

#### 3.2.4. Follow-Up

Follow-up on the 45th postoperative day revealed no significant pathological findings.

### 3.3. Case 3

#### 3.3.1. Presentation

A 49-year-old male with a history of gastroduodenal ulcer disease was referred to our department with sudden epigastric pain and vomiting after excessive alcohol ingestion four hours before presentation. The clinical picture was associated with respiratory distress and anxiety. At presentation we found 105/87 a blood pressure, a regular heart rate of 99 beats per minute, a body temperature of 37.7 °C, a respiratory rate of 32 breaths per minute, and an oxygen saturation level of 82%. His height and weight were 168 cm and 83.1 kg, respectively.

#### 3.3.2. Diagnosis

Pneumothorax with spontaneous rupture of the esophagus was diagnosed using CT imaging. The patient was admitted to the ICU for hemodynamic monitoring and respiratory support, and emergency surgery was performed.

#### 3.3.3. Operative Details

**Figure 5:**
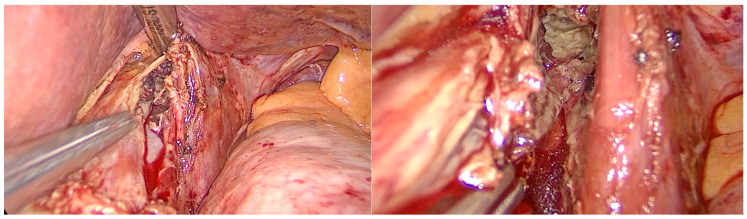
Intraoperative aspect of laparoscopic Boerhaave’s syndrome. **Left** image: perforation in the left wall of the distal esophagus. **Right** image: left crus of the diaphragm and pleural cavity with necrotic tissue.

We performed a laparoscopic transhiatal approach, esophageal perforation suture, and pleural drainage (Figure 5). The postoperative course was uneventful, and the patient was discharged on the 12th postoperative day.

#### 3.3.4. Follow-Up

On the 30th postoperative day, follow-up upper endoscopy revealed a favorable outcome with a normal aspect of the esophageal mucosa.

## 4. Discussion

On a global scale, the annual incidence of BS is approximately 3.1 cases per million individuals; however, this number is likely underreported because of diagnostic challenges and misclassification [3].

The primary mechanism most often associated with the development of BS is forceful emesis, which results in an abrupt increase in intraesophageal pressure. This pressure surge is attributed to a disruption in neuromuscular coordination between the upper and lower esophageal sphincters, compounded by the failure of the cricopharyngeal muscle to relax appropriately [4,7,8]. In approximately 80% of cases, elevated intraesophageal pressure leads to full-thickness tear of the distal esophageal wall.

### 4.1. Diagnosis and Imaging

Its signs and symptoms are often nonspecific, which can complicate the diagnostic process and delay appropriate interventions. The classic clinical triad described by Mackler—vomiting, chest discomfort, and subcutaneous emphysema—is not consistently present and occurs in only approximately half of the cases; diagnostic certainty tends to improve substantially as more features of the triad become apparent in the patient presentation [14,15,16]. Beyond the classical manifestations, a range of atypical and nonspecific symptoms has been documented, which may contribute to diagnostic ambiguity. Reported presentations include hematemesis, retrosternal burning suggestive of heartburn [7], acute hypoxemic respiratory failure [8], and abdominal tenderness [6,7]. Nevertheless, forceful vomiting remains the predominant clinical feature in most cases. Delayed diagnosis owing to nonspecific symptoms is common; however, imaging results can establish a definitive diagnosis.

In approximately 90% of cases, a contrast esophagogram can confirm the diagnosis, typically employing a water-soluble contrast medium to minimize the risk of barium leakage and associated complications such as inflammation within the pleural or mediastinal spaces. The sensitivity of this imaging approach varies depending on the characteristics of the perforation, such as its size and anatomical location, as well as the specific technique applied. Esophagography with water-soluble contrast, such as gastrografin, is 90% sensitive, and research indicates that false-negative rates can occur in 10–38% of cases [17].

Contrast-enhanced CT is a valuable diagnostic tool that offers detailed visualization of the rupture site and the presence of drainable fluid collections in the thoracic or abdominal cavities. CT findings may include esophageal wall thickening and edema, fluid accumulation in the pleural or retroperitoneal spaces, and mediastinal widening [17,18].

Regarding differential diagnosis, other thoracic and abdominal disorders that can mimic BS should be excluded, including perforated peptic ulcer, spontaneous pneumothorax, pancreatitis, aortic dissection, pericarditis, pneumonia, Mallory–Weiss tear, pulmonary embolus, and myocardial infarction [19].

### 4.2. Role of Endoscopic Intervention/Stent Placement

In the therapeutic management of this condition, endoscopic intervention performed by skilled endoscopists represents an alternative approach to surgery [20]. The purpose of stent placement is to cover the site of esophageal rupture to prevent superinfection and create suitable conditions for healing. However, the main limitation of this method, as reported in several observational studies, is that a considerable number of patients undergoing endoscopic treatment ultimately require subsequent surgical intervention due to treatment failure or complications [21,22]. In recent years, both endoscopic and conservative approaches have been explored as potential treatment options for this condition [20]. Nevertheless, several studies have indicated that conservative management, particularly stent placement, is associated with higher mortality rates than surgical intervention in some instances. In addition, available evidence indicates that stent-based therapy carries a higher risk of mortality than primary surgical intervention [23]. Subsequent evaluations have indicated that although initial closure of the esophageal defect with stent placement can be achieved successfully, a considerable number of patients later develop secondary complications, including pleural empyema and mediastinitis, which frequently necessitate additional surgical intervention.

The rate of therapeutic failure following endoscopic stenting remains notably elevated, with a significant proportion of patients ultimately requiring definitive surgical intervention to achieve adequate source control of the disease. Furthermore, current evidence does not demonstrate clear benefits of endoscopic stenting in terms of the duration of intensive care or hospital stay or in reducing the incidence of sepsis or multiple organ dysfunction when compared with primary surgical repair [22].

Tellechea et al. concluded that endoscopy can be a useful tool at all stages of BS management: difficult diagnosis, primary treatment in selected patients, and salvage when surgery fails [24]. Moreover, endoscopic stenting in combination with thoracoscopic debridement has been reported to be an effective and safe hybrid approach with low mortality rates [25]. A recent retrospective analysis demonstrated that endoscopic vacuum therapy (EVT) achieved a notably high rate of therapeutic success in the management of BS, suggesting its potential as an effective treatment modality [26].

### 4.3. Surgical Management

Some studies have emphasized that postponing adequate therapeutic intervention beyond the initial 24 h window from symptom onset is strongly correlated with increased mortality, underscoring the critical importance of early diagnosis and timely management in improving patient survival [27]. In contrast, some authors have reported successful primary repair in late presentations (after 48 h) with promising results [13,28]. Our experience with BS is related only to early onset symptoms; hence, there was a short delay in initiating specific treatment.

In early spontaneous esophageal perforation, surgical repair and chest drainage are the standard of care with the lowest morbidity and mortality [29,30]. 

In patients with early diagnosed perforations (up to 24 h) and hemodynamically stable patients, minimally invasive techniques can achieve good results.

Early surgical management is indicated to achieve the best possible outcome. The anatomical site of the esophageal tear significantly influences both the clinical manifestations and the spectrum of potential complications. When a rupture occurs within the thoracic segment, there is a risk of gastric contents leaking into the surrounding structures, potentially resulting in mediastinitis, subcutaneous or mediastinal emphysema, empyema, or even tissue necrosis. Lesions located in the upper or mid-thoracic esophagus are more frequently associated with the development of hydropneumothorax or right-sided pleural effusion [15,16].

The standard surgical treatment is open thoracotomy and/or laparotomy; however, there are many reports of successful thoracoscopic and laparoscopic management [31,32,33]. Several authors advocate for primary defect closure in conjunction with adequate drainage, emphasizing that the therapeutic strategy should be tailored to patient-specific characteristics, such as advanced age [34].

#### 4.3.1. Role of Thoracoscopic Approach

The thoracoscopic approach is indicated for middle thoracic esophageal rupture, achieving good results, but only in stable patients; this approach is to be used when expertise is available. The advantages of this approach are multiple: magnification is superior to that of conventional thoracotomy, minimal blood loss, reduced postoperative trauma, and easy conversion to thoracotomy if necessary [35,36].

#### 4.3.2. Role of Laparoscopic Approach

In the distal esophagus, the transabdominal approach for esophageal tear is safe, and the laparoscopic technique is recommended. However, the available literature on laparoscopic transhiatal BS repair remains scarce. In 2002, Landen and El Nakadi reported a small pilot series involving three patients who underwent laparoscopic surgical repair of Morgagni hernia. Of these, two experienced favorable postoperative recovery, whereas one developed severe complications, leading to multiple organ dysfunction and death [37].

Subsequent publications have described additional cases of Boerhaave’s syndrome managed exclusively through a laparoscopic transhiatal approach; all patients recovered with no mortality, and the postoperative outcomes were consistently favorable [38,39,40]. Mikamy et al. presented a hand-assisted laparoscopic procedure for esophageal rupture [40].

Minimally invasive techniques allow adequate esophageal repair, as depicted in our case series. In our cases, we used continuous sutures of resorbable materials. The use of continuous or interrupted sutures can have the same outcome, but this also depends on the quality of the tissue [11,38,39].

#### 4.3.3. Role of Video-Assisted Thoracoscopic Surgery (VATS)

One of the minimally invasive techniques is video-assisted thoracoscopic surgery (VATS), which can replace open surgery, as some authors have claimed [41]. Haveman et al. demonstrated that VATS outcomes are comparable to those of open thoracotomy in achieving effective mediastinal and pleural drainage in patients with Boerhaave syndrome. In their prospective series, only 2 of 12 patients required conversion to thoracotomy, while postoperative morbidity was lower than that in the historical open-surgery cohort [42]. These findings indicate that VATS can be a safe and effective therapeutic alternative, offering reduced surgical trauma, shorter recovery time, and decreased complication rates, particularly in high-risk or fragile patients.

### 4.4. Case Series Considerations and Perspectives

In the present cases, timely diagnosis and appropriate surgical management prevented the evolution of severe septic complications, confirming the importance of early multidisciplinary intervention in this rare but critical condition. These three cases highlight the importance of rapid diagnosis and early surgical intervention in BS, demonstrating the successful management of severe conditions through multidisciplinary efforts [43,44].

Moreover, a laparoscopic surgical approach aids in this effort [45]. One patient underwent surgery 5 days after admission, emphasizing the possibility of preoperative planning in certain cases. Conservative measures, such as nil oral intake, intravenous fluids, antibiotics, nasogastric suction, and chest drainage, can prolong the time until the right expertise and facilities are available, as Andrew et al. pointed out [46]. As was pointed out in the paper, successful primary repair can be obtained in early or delayed presentation; this depends on the tissue necrosis and edema that can be present.

## 5. Conclusions

The treatment of patients with spontaneous esophageal rupture is a surgical challenge, with no standard treatment established. In selected early, hemodynamically stable patients with distal tears and limited contamination, laparoscopic transhiatal with drainage is a safe and feasible option. Our findings suggest that the laparoscopic approach is a safe and viable alternative, offering the advantage of reduced hospitalization time compared with cases managed via the thoracic route.

Importantly, this minimally invasive technique can be readily integrated into surgical practice at centers already experienced in treating conditions of the gastroesophageal junction, thereby facilitating its broader application. Nevertheless, multicenter randomized studies are needed to establish this practice.

## Figures and Tables

**Table 1:** Summary of characteristics for Boerhaave syndrome patients in the study (CT—computed tomography; TPN—total parenteral nutrition).

Patient No.	Age/Sex	Onset Symptoms (h)	ASA Class	Imaging	Day of Surgery (Day from Admission)	Surgical Approach	Operative Time (min)	Length of Stay (Days)	Follow-Up (Postoperative Day)	Supplementary Procedures
1	54 yrs/m	6 h	II	CT + oral contrast	1st	Laparoscopic + mediastinal drainage	220	19	60th	Feeding enterostomy
2	38 yrs/m	8 h	I	CT + oral contrast	5th	Laparoscopic + mediastinal drainage	180	14	45th	TPN
3	49 yrs/m	4 h	I	CT + oral contrast	1st	Laparoscopic + mediastinal drainage	190	12	30th	TPN

## Data Availability

Not applicable.

## Abbreviations

The following abbreviations are used in this manuscript:

BS	Boerhaave syndrome
CT	Computed tomography
TPN	total parenteral nutrition
VATS	video-assisted thoracoscopic surgery
